# Molecular evolution of HIV-1 integrase during the 20 years prior to the first approval of integrase inhibitors

**DOI:** 10.1186/s12985-017-0887-1

**Published:** 2017-11-14

**Authors:** Karolin Meixenberger, Kaveh Pouran Yousef, Maureen Rebecca Smith, Sybille Somogyi, Stefan Fiedler, Barbara Bartmeyer, Osamah Hamouda, Norbert Bannert, Max von Kleist, Claudia Kücherer

**Affiliations:** 1 HIV and other Retroviruses, Robert Koch Institute, Berlin, Germany; 2 Department of Mathematics and Computer Science, Freie Universität Berlin, Berlin, Germany; 3 HIV/AIDS, STI and Blood-borne Infections, Robert Koch Institute, Berlin, Germany

**Keywords:** HIV, Integrase, Drug resistance, Polymorphisms, Time trend, Covariation

## Abstract

**Background:**

Detailed knowledge of the evolutionary potential of polymorphic sites in a viral protein is important for understanding the development of drug resistance in the presence of an inhibitor. We therefore set out to analyse the molecular evolution of the HIV-1 subtype B integrase at the inter-patient level in Germany during a 20-year period prior to the first introduction of integrase strand inhibitors (INSTIs).

**Methods:**

We determined 337 HIV-1 integrase subtype B sequences (amino acids 1–278) from stored plasma samples of antiretroviral treatment-naïve individuals newly diagnosed with HIV-1 between 1986 and 2006. Shannon entropy was calculated to determine the variability at each amino acid position. Time trends in the frequency of amino acid variants were identified by linear regression. Direct coupling analysis was applied to detect covarying sites.

**Results:**

Twenty-two time trends in the frequency of amino acid variants demonstrated either single amino acid exchanges or variation in the degree of polymorphy. Covariation was observed for 17 amino acid variants with a temporal trend. Some minor INSTI resistance mutations (T124A, V151I, K156 N, T206S, S230 N) and some INSTI-selected mutations (M50I, L101I, T122I, T124 N, T125A, M154I, G193E, V201I) were identified at overall frequencies >5%. Among these, the frequencies of L101I, T122I, and V201I increased over time, whereas the frequency of M154I decreased. Moreover, L101I, T122I, T124A, T125A, M154I, and V201I covaried with non-resistance-associated variants.

**Conclusions:**

Time-trending, covarying polymorphisms indicate that long-term evolutionary changes of the HIV-1 integrase involve defined clusters of possibly structurally or functionally associated sites independent of selective pressure through INSTIs at the inter-patient level. Linkage between polymorphic resistance- and non-resistance-associated sites can impact the selection of INSTI resistance mutations in complex ways. Identification of these sites can help in improving genotypic resistance assays, resistance prediction algorithms, and the development of new integrase inhibitors.

**Electronic supplementary material:**

The online version of this article (10.1186/s12985-017-0887-1) contains supplementary material, which is available to authorized users.

## Background

The HIV-1 integrase catalyses the integration of the reverse transcribed viral DNA into the host genomic DNA via a two-step process. In its active form the integrase forms a tetramer. The monomeric enzyme consists of 288 amino acids (aa) and contains three functional domains: the N-terminal zinc-binding domain (NTD, aa 1–49), the central catalytic core domain (CCD, aa 50–212), and the C-terminal DNA-binding domain (CTD, aa 213–288) [1–4]. Each region comprises motifs essential for the proper function of the enzyme, e.g. the zinc finger motif H12-H16-C40-C43 in the NTD, the active site D64-D116-E153 in the CCD, and the minimal nonspecific DNA-binding region ranging from I220 to D270 in the CTD [2–6].

Raltegravir was the first integrase strand inhibitor (INSTI) to be approved in Europe in 2007, followed by elvitegravir in 2012 and dolutegravir in 2014. Bictegravir [7] and cabotegravir [8] are in clinical trial development. INSTIs target the CCD, thereby inhibiting the strand transfer of the double-stranded viral DNA into the host genome [1]. Various allosteric inhibitors of integrase (ALLINIs), which modulate integrase multimerisation [9] and interfere with the cellular transcription factor LEDGF/p75 [10] are in development but have not so far made it further than Phase I clinical trial [11].

Due to its high rate of replication, mutation, and recombination, HIV is a virus of high genetic variability. The viability of virus variants in turn is limited by structural and functional constraints. At the same time, variants in the viral quasispecies can be selected by the pressure of the human immune system [12, 13] or antiretroviral treatment (ART) [14, 15]. In general, the HIV diversity at the intra-patient level increases during the course of infection [16] driven by both drift and selection [17]. During transmission to a new host, several stochastic and selective bottlenecks reduce the viral diversity to a few variants [18], and factors that contribute to shaping the HIV diversity at the inter-patient level are extensively discussed [19–24].

Naturally occurring polymorphisms can affect the genetic barrier to drug resistance by influencing the selection of resistance mutations, enzymatic activity, and replicative capacity [22, 25, 26]. Epistatic interactions between polymorphisms can further modulate viral fitness and the development of drug resistance in complex ways and have been shown to play an important role in the HIV-1 protease and reverse transcriptase [27–30]. Thus, to understand the selection of resistant variants in the presence of INSTIs, it is important to investigate the evolutionary dynamics of the polymorphic sites in the integrase.

The prevalence of HIV-1 integrase polymorphisms and INSTI resistance mutations has been investigated before in INSTI-naïve individuals [26, 31–35] and ART-naïve individuals [35–40]. However, time trends and covariation of complex mutation patterns preceding the availability of INSTIs have not so far been analysed. The aim of this study was to investigate covarying clusters of naturally occurring resistance- and non-resistance-associated amino acid variants and their frequencies over time at the inter-patient level to consider their potential relevance for INSTI resistance. To this end, HIV-1 integrase sequences were obtained from samples of ART-naïve individuals newly diagnosed with HIV-1 between 1986 and 2006, a 20-year period prior to the first approval of INSTI in Germany [41].

## Methods

### Study population

Plasma samples from individuals newly diagnosed with HIV-1 between 1986 and 1996 (*N* = 167) were archived at the former diagnostic unit of the National AIDS Centre in Germany and stored at −40 °C. The date of HIV-1 diagnosis is the same as the plasma sampling date for the HIV-1 integrase genotyping. Plasma samples from individuals newly diagnosed with HIV-1 between 1997 and 2006 (*N* = 170) were collected for the German HIV-1 Seroconverter Study [42–45] and stored at −70 °C. These plasma samples were taken within 12 months after diagnosis. In total, the study population comprised 337 individuals (Table 1).

**Table 1:** Number of HIV-1 integrase sequences per year/period of HIV-1 diagnosis

Year of diagnosis	No. of HIV-1 sequences/year	No. of HIV-1 sequences/period
1986	36	84
1987	3	
1988	17	
1989	28	
1990	0	58
1991	3	
1992	30	
1993	5	
1994	20	
1995	18	42
1996	7	
1997	7	
1998	10	
1999	7	49
2000	10	
2001	12	
2002	20	
2003	17	104
2004	22	
2005	24	
2006	41	

### HIV-1 integrase genotyping

Viral RNA from 500 μl plasma was pelleted by centrifugation (20,800 g, 90 min, 4 °C) and then isolated using the Viral RNA Mini Kit (Qiagen, Germany) according to the manufacturer’s instructions. Reverse transcription and polymerase chain reaction were performed using the OneStep RT-PCR kit (Qiagen, Germany) with primers 5′-INT (5′- ATT GGA GGA AAT GAA CAA GT -3′; nucleotides (nt) 4173–4192, Acc. K03455) and 3p31as (5′- ATC CTG TCT ACY TGC CAC ACA A -3′; nt 5066–5087, Acc. K03455) [37]. Amplicons were purified (QIAquick spin PCR purification kit, Qiagen, Germany) and sequenced by cycle-sequencing (ABI Big Dye 3.1, Gene Amp Applied Biosystems PCR System 9700, Thermo Fisher Scientific, Germany) with the primers listed above and the additional primers F2 s (5′- TAA GAC AGC AGT ACA AAT GGC AG -3′; nt 4745–4767, Acc. K03455) and F3as (5′- GCT GTC CCT GTA ATA AAC CCG -3′; nt 4899–4919, Acc. K03455). Sequencing was performed on an ABI Prism 310 capillary sequencer (Thermo Fisher Scientific, Germany), and SeqMan Pro (Lasergene v10.0.1, DNASTAR, USA) was used for sequence analysis. This genotyping assay had a detection limit of 10^3^ copies/ml for HIV-1 subtype B and yielded a 915 bp amplicon spanning HIV-1 integrase bp 1–278. Only subtype B strains were included in the analyses. The HIV-1 subtype was determined using the REGA HIV Subtyping Tool (http://dbpartners.stanford.edu:8080/RegaSubtyping/stanford-hiv/typingtool/).

### INSTI resistance mutations

Major INSTI resistance mutations (T66I, E92Q, F121Y, Y143CHR, S147G, Q148HKR, N155H) that confer substantial phenotypic resistance to at least one of the currently approved INSTI as well as minor INSTI resistance mutations (T66AK, L74 M, E92G, T97A, E138AK, G140AS, R263K) that increase INSTI resistance and/or viral replication capacity were identified according to the IAS-list [46]. In addition, the following minor INSTI resistance mutations according to the current definitions of the resistance prediction algorithm HIVdb (http://hivdb.stanford.edu, version March 2, 2017), ANRS (http://www.hivfrenchresistance.org, version no. 26, September 2016), HIV-GRADE (http://www.hiv-grade.de, version January 16, 2017), and Rega (https://rega.kuleuven.be/cev/avd/software/rega-algorithm, v9.0.1, October 29, 2013) were considered: A49G, H51Y, V54I, L68IV, L74I, E92V, Q95K, H114Y, G118R, S119R, T124A, A128T, E138T, G140C, Y143AGS, P145S, Q146IKLPR, Q148EG, V151AIL, S153FY, N155ST, K156 N, E157Q, G163KR, T206S, S230GNR, D232N, V260I. Moreover, INSTI-selected mutations that were observed in vitro or in vivo were investigated [47, 48]: M50I, G59E, I60L, I72A, Q95T, L101IY, T112S, F121Y, T122I, T125A, E138D, Y143K, Q148N, M154I, I162M, G163E, Q177R, G193E, V201I, I203M, I204T.

### Assessment of phylogenetic bias

A unique anonymising code provided with the mandatory report of newly HIV-1 infected cases to the Robert Koch-Institute ensured that only one HIV-1 sequence per patient was included in our dataset. However, to investigate whether our results were biased by the overrepresentation of phylogenetically closely related sequences (i.e. sequences originating from direct transmission events) we identified clusters of sequences with a very small phylogenetic distance and replaced them with one representative sequence (the sequence from the patient who was diagnosed first). For clustering, a multiple sequence alignment (MSA) including an HIV-1 group N reference sequence (Acc. AJ006022) was generated with clustalW (BioEdit, v7.2.5, Tom Hall) and end-trimmed to nt 4230–5064 (Acc. K03455). We computed a maximum likelihood phylogeny with 500 times nonparametric bootstrap with replacement using the program RAxML [49]. The HIV-1 group N sequence was used to root the maximum likelihood tree. Applying the program Transmic [50], a set of phylogenetically closely related sequences was identified if the mean of all pairwise patristic distances did not exceed a threshold of 0.015 expected nucleotide substitutions per site and the most recent common ancestor node had a bootstrap support of 0.9 [50, 51]. We detected 11 clusters, each comprising two sequences. The final reduced dataset consisted of 326 sequences. All analyses were then performed with the full dataset and the reduced dataset to assess any potential bias. All reported results were confirmed on the basis of both datasets while all numbers reported in the results section are given for the full dataset.

### Amino acid variability

MSAs for the full (337 sequences) and the reduced (326 sequences) dataset were generated with clustalW (BioEdit, v7.2.5, Tom Hall) and end-trimmed to nt 4230–5064 (Acc. K03455). The nucleotide sequences were then translated into amino acid sequences. Nucleotide sequence ambiguities of codons were resolved during translation. An X was assigned if multiple amino acids resulted from the translation of codons containing nucleotide sequence ambiguities to avoid consideration of amino acid variants only present in mixtures due to PCR and sequencing errors. The frequencies of amino acids at positions 1 to 278 of the HIV-1 integrase were calculated. A position was defined to be polymorphic if, on the basis of the full and the reduced dataset at this position, a total of 1% or more amino acid variants were present when compared to the consensus B sequence (http://hivdb.stanford.edu/pages/documentPage/consensus_amino_acid_sequences.html). All other positions were defined as conserved.

We also analysed the Shannon entropy *E*_*i*_, which quantifies the degree of variability at a single amino acid position *i* ∊ [1,278], according to

$$ \kern13.5em {\mathit{\mathsf{E}}}_{\mathit{\mathsf{i}}}=-\sum \limits_{\mathit{\mathsf{x}}}\ \mathit{\mathsf{P}}\left({\mathit{\mathsf{X}}}_{\mathit{\mathsf{i}}}=\mathit{\mathsf{x}}\right){\mathit{\mathsf{\log}}}_{\mathsf{2}}\mathit{\mathsf{P}}\left({\mathit{\mathsf{X}}}_{\mathit{\mathsf{i}}}=\mathit{\mathsf{x}}\right) $$

where *P*(*X*_*i*_ = *x* ) denotes the probability of observing the particular amino acid *x* at position *i* [52]. A high value of entropy *E*_*i*_ indicates high amino acid variability at position *i*.

### Amino acid time trends

The samples were grouped into five time periods (1986–1989, 1990–1994, 1995–1998, 1999–2002, and 2003–2006) to obtain a more homogenous distribution and to render the time trend analyses more robust (Additional file 1: Table S1). Next, we calculated the frequencies of all amino acids at positions 1 to 278 of the HIV-1 integrase within the periods. We then fitted a linear regression by minimizing the sum of least square deviation between detected and predicted frequencies *y* and *f*(*a*, *b*) for each period *i* according to

$$ \left\{\mathit{\mathsf{a}},\mathit{\mathsf{b}}\right\}=\mathit{\mathsf{a}\mathsf{rgmin}}\sum \limits_{\mathit{\mathsf{i}}}{\left(\mathit{\mathsf{f}}\left(\mathit{\mathsf{a}},\mathit{\mathsf{b}},{\mathit{\mathsf{x}}}_{\mathit{\mathsf{i}}}\right)-\mathit{\mathsf{y}}\left({\mathit{\mathsf{x}}}_{\mathit{\mathsf{i}}}\right)\right)}^{\mathsf{2}} $$

with *f*_*i*_(*a*, *b*, *x*_*i*_) = *a* ∙ *x*_*i*_ + *b* where *a* denotes the slope and *b* the intersect of the linear function. The variable *x*_*i*_ denotes the distance of the centre of period *i* [in years] to the centre of the first period. In order to detect significant time trends, we generated 10,000 bootstrap samples by drawing sequences from the original sequence set with replacement. For each resampled set, we computed mutation frequencies and performed the linear regression as described above. Raw *P* values were then computed from the bootstrap distribution of fitted slopes *a* in analogy to Katchanov et al. [53], i.e. we computed ${P}^{+}=\frac{\#a\le 0.001}{10000}$ to test whether the frequency of that mutation is significantly increasing by at least 0.1% per year (*H*_0_ : *a* ≤ 0.001 vs. *H*_1_ : *a* > 0.001) and conversely ${P}^{-}=\frac{\#a\ge -0.001}{10000}$ to assess whether it is significantly decreasing by at least 0.1% per year (*H*_0_ : *a* ≥  − 0.001 vs. *H*_1_ : *a* <  − 0.001). *P* values (*P* = min(*P* ^−^, *P* ^+^)) were subsequently corrected for multiple testing using the false discovery rate (FDR) method by Benjamini-Hochberg [54]. Time trends were considered significant if they were identified on the basis of the full and the reduced dataset with a FDR corrected *P* < 0.05.

### Amino acid covariation

In order to determine if amino acid positions are covarying, we applied direct coupling analysis where direct correlations are disentangled from transitive correlations. We computed evolutionary coupling terms ec_*ij*_ using the recently developed plmc tool (https://github.com/debbiemarkslab/plmc) [55]. This tool infers couplings by fitting a Potts model to the MSA using a pseudo likelihood approach with L_2_ regularisation. We used default regularisation parameters of λ_1_ = 0.01 and λ_2_ = 100 for single site contributions and direct couplings terms *e*_ij_(α,β), respectively. To correct for a phylogenetic bias by reweighting neighbouring sequences, we chose the default parameter θ = 0.01. Ambiguous amino acids (X) were discarded during inference. As expected, the inferred coupling terms are approximately normally distributed with mean 0 and standard deviation 1/λ_2_.

The direct coupling terms *e*_*ij*_(*α,β*) describe the direct correlation of two amino acids *α* and *β* at positions *i* and *j*. The overall interaction of two positions *i* and *j* are given by the evolutionary coupling term *ec*_*ij*_, which is the Frobenius norm of the direct coupling terms

$$ \parallel {\mathit{\mathsf{e}}}_{\mathit{\mathsf{ij}}}{\parallel}_{\mathsf{2}}=\sqrt{\sum \limits_{\mathit{\mathsf{\alpha}},\mathit{\mathsf{\beta}}}{\mathit{\mathsf{e}}}_{\mathit{\mathsf{ij}}}{\left(\mathit{\mathsf{\alpha}},\mathit{\mathsf{\beta}}\right)}^{\mathsf{2}}} $$

with average product correction (APC), to suppress phylogenetic bias effects [56]:

$$ {ec}_{ij}=\parallel {e}_{ij}{\parallel}_2-\frac{\parallel {e}_{i\ast }{\parallel}_2\bullet \parallel {e}_{\ast j}{\parallel}_2}{\parallel {e}_{\ast \ast }{\parallel}_2}. $$

To identify significant coupling terms, z-scores were computed according to

$$ {Zscore}_{ij}=\frac{e_{ij}-\mu \left({e}_{\ast \ast}\right)}{\sigma \left({e}_{\ast \ast}\right)} $$

where *μ*(*e*_∗∗_) and *σ*(*e*_∗∗_) denote the mean and standard deviation of the estimated evolutionary coupling terms between all position pairs *i* < *j*. Z-scores were then converted into *P* values and corrected for multiple testing using the Benjamini-Hochberg method [54]. We used *P* < 0.005 to detect significant couplings [57], which corresponds to a Bayes factor of 14 typically used in Bayesian inference [58].

To ensure the robustness of the statistical inference, Halsey et al. [59] recently proposed to combine power analysis with *P* value based statistical analysis. In line with these recommendations, we generated 1000 MSAs by sampling sequences with replacement from the original alignment. For each resampled MSA, we inferred the coupling terms and performed the statistical tests as outlined above. In line with Halsey’s recommendations, we only reported coupling terms that were significant in at least 95% of the resamplings, i.e. where the respective test power was >0.95. An interactive plot to explore the detailed results of the direct coupling analysis was generated using the EVZoom tool (https://github.com/debbiemarkslab/EVzoom) and can be accessed through http://page.mi.fu-berlin.de/msmith/couplings_integrase.html.

## Results

### Characteristics of the study population

All 337 individuals were newly diagnosed with HIV-1, ART-naïve, and infected with an HIV-1 subtype B strain. The median age at HIV-1 diagnosis was 32 (inter-quartile range 27–38) years. Most individuals were male (297/337, 88.1%), 11.0% (37/337) were female, and for 0.9% (3/337) the gender was not documented. The most common transmission group was men having sex with men (220/337, 65.3%), followed by 14.8% heterosexual transmission (50/337) and 14.2% injection drug users (48/337). For 5.6% (19/337) of the individuals the risk factor for transmission was unknown.

### Frequency of INSTI resistance mutations

As expected, no major INSTI resistance mutations were detected in this ART-naïve study population from the period prior to INSTI release. Moreover, all major INSTI resistance-associated positions were fully conserved, and amino acids at these positions corresponded to the respective INSTI-sensitive consensus B amino acids. However, some minor INSTI resistance mutations and INSTI-selected mutations were identified (Table 2).

**Table 2:** Overall frequency (%) of minor INSTI resistance mutations and INSTI-selected mutations

	Overall frequency (%) of	
Variant	minor INSTI resistance mutation	INSTI-selected mutation
M50I		12.8
V54I	0.9	
G59E		2.1
L68V	1.2	
L74I	2.7	
L74 M	0.6	
L101I		36.8
S119R	3.0	
T122I		24.3
T124A	25.8	
T124 N		
T125A		17.8
A128T	0.9	
E138D		1.5
V151A	0.3	
V151I	7.4	
M154I		18.4
K156 N	5.3	
E157Q	2.4	
G163E		2.4
G163R	0.3	
V165I		
H171Q		
G193E		10.1
V201I		36.8
I203M		3.3
T206S	6.8	
N222 K		
S230G	0.6	
S230 N	8.0	
V260I	0.3	

### Amino acid variability

**Fig. 1:**
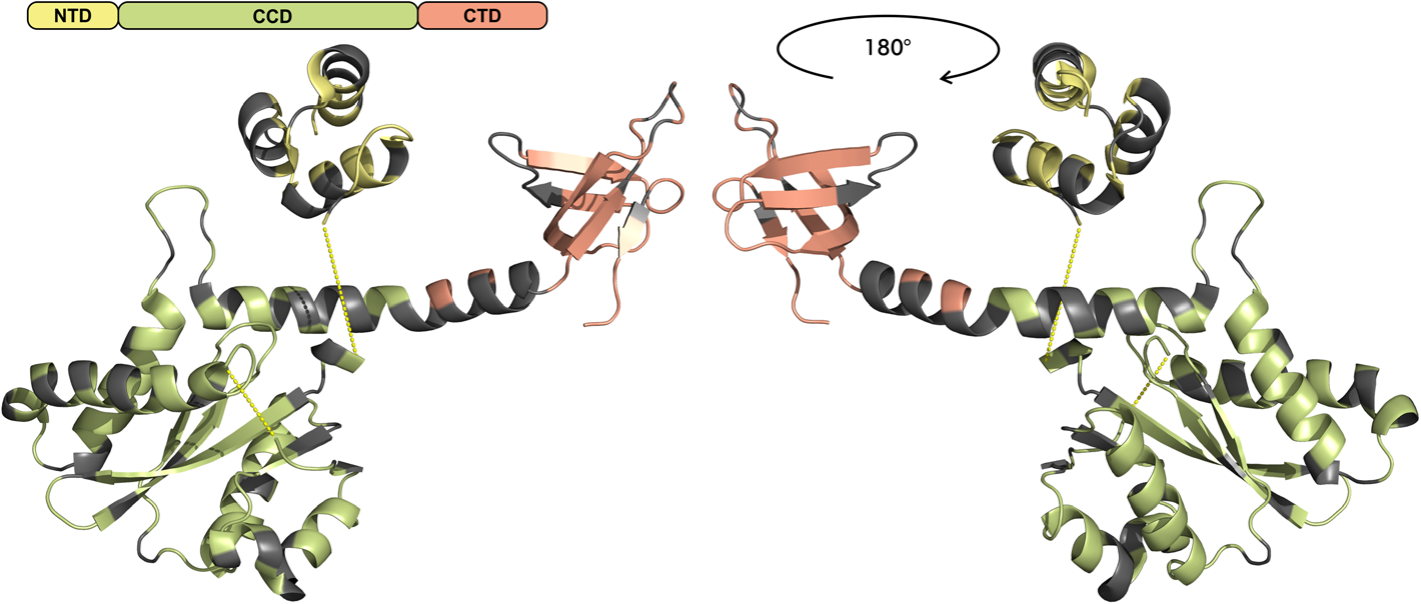
Ribbon model of the HIV-1 integrase monomer from two viewpoints. The model was constructed in PyMol [67] as an overlay of protein database structures 1ex4 [3] and 1k6y [4]. The NTD (aa 1–49) is coloured yellow, the CCD (aa 50–212) green, and the CTD (aa 213–288) red. Polymorphic sites are coloured grey

**Fig. 2:**
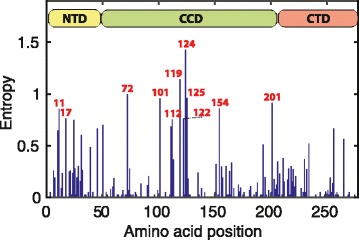
Levels of Shannon entropy at amino acid positions 1–278 of the HIV-1 integrase. Positions with entropy levels >0.75 and the three functional domains of the integrase are indicated

Eighty-six polymorphic sites (30.9%) were observed within the 278 amino acid positions examined in the HIV-1 integrase. Proportionately, most polymorphic positions were found in the NTD (20/49, 40.8%). There were equal proportions of polymorphic sites within the CCD (47/163, 28.8%) and the CTD (19/66, 28.8%) (Fig. 1). The highest amino acid variability with entropies >0.75 was found in the NTD at positions 11 and 17 as well as in the CCD at positions 72, 101, 119, 122, 124, 125, 154, and 201 (Fig. 2).

The zinc finger motif (H12-H16-C40-C43), the active site (D64-D116-E153), the multimerisation motif (K186-R187-K188), and most sites interacting with the cellular cofactor LEDGF/p75 or within the minimal nonspecific DNA binding region were conserved. Nevertheless, positions 125, 165, 167, 171, 172, and 173, involved in binding LEDGF/p75, were polymorphic with entropies up to 0.96. Likewise, within the minimal nonspecific DNA binding region positions 220–222, 227, 230, 232, 234, 251–256, and 265 exhibited entropies up to 0.66 (Fig. 2).

### Amino acid time trends

**Fig. 3:**
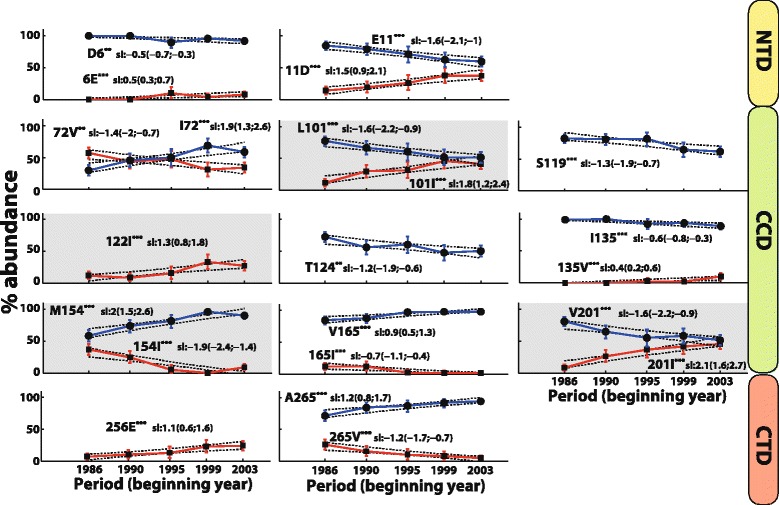
Amino acid variants with significant trends in frequency over time. The median frequencies of variants corresponding to the consensus B reference sequence within each period are indicated by filled circles with blue error bars indicating the 5th–95th percentile ranges. The median frequencies of variants differing from the consensus B reference sequence within each period are indicated by filled squares with red error bars indicating the 5th–95th percentile ranges. The median predicted slopes are depicted in blue or red solid lines. The 5th–95th percentile ranges of the model-predicted trends are indicated by dotted lines. Predicted slopes sl are given in units %/year. Positions are grouped by the three functional domains of the integrase. Positions showing trends in INSTI resistance-associated variants are shaded grey. *** time trend is significant at the *P* < 0.01 level, ** time trend is significant at the *P* < 0.05 level

Twenty-two significant time trends in amino acid frequencies were discovered at 13 polymorphic positions. Proportionally, most of these sites were located in the CCD (9/163, 5.5%), followed by location in the NTD (2/49, 4.1%) and the CTD (2/66, 3.0%) (Fig. 3).

At nine positions (6, 11, 72, 101, 135, 154, 165, 201, 265) an amino acid exchange between two variants was identified. In contrast, at sites 119 and 124 increasing polymorphy was observed since variants decreased in frequency without being replaced by another variant. Finally, variants 122I and 256E increased in frequency without replacing another variant, although a decrease of T122 and D256 was observed that was close to statistical significance (Fig. 3).

Variants L101I, T122I, and V201I with increasing frequency as well as M154I with decreasing frequency were associated with INSTI resistance (Fig. 3).

### Amino acid covariation

**Fig. 4:**
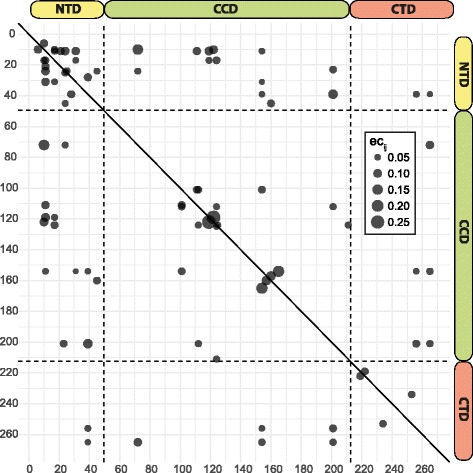
Significant couplings within the HIV-1 integrase. The size of the dots indicate the values of the evolutionary coupling terms ec*ij* and are plotted mirrored for position *i* against position *j*. The dashed horizontal and vertical lines seperate the three functional domains of the integrase

Overall, 42 significant couplings were identified (Table 3). 10 couplings appeared within the NTD, 10 within the CCD, and two within the CTD. 13 couplings were observed between the NTD and the CCD, five between the CCD and the CTD, and two between the NTD and the CTD. In total, 25 and 28 couplings involved at least one position within the NTD and the CCD, respectively (Fig. 4).

**Table 3:** Evolutionary coupling terms for covarying positions within the HIV-1 integrase

position *i*	position *j*	ec*ij*
6	10	0.0823
10	17	0.0623
10	72	0.1550
10	122	0.0994
11	17	0.0668
11	21	0.0847
11	24	0.0924
11	31	0.0883
11	111	0.0858
11	119	0.0995
11	154	0.0500
17	31	0.0516
17	119	0.0562
17	124	0.0755
23	201	0.0721
24	25	0.0717
24	45	0.0543
24	72	0.0554
28	39	0.0826
31	154	0.0362
39	154	0.0449
39	201	0.1201
39	256	0.0559
39	265	0.0411
45	160	0.0757
72	265	0.0897
101	111	0.0738
101	112	0.0677
101	154	0.0740
112	124	0.0567
112	201	0.0612
119	122	0.2792
124	125	0.0466
124	211	0.0589
154	165	0.1792
154	256	0.0488
154	265	0.0647
157	160	0.1177
201	256	0.0781
201	265	0.0641
219	222	0.0730
234	253	0.0679

Evolutionary coupling terms ec*ij* between position *i* and *j* with *P* < 0.005 and power > 0.95 are given

The LEDGF/p75-binding sites 125 and 165 were observed in one coupling each. Nine couplings involved sites 222, 234, 253, 256, or 265 of the minimal nonspecific DNA-binding region (Table 3).

17 amino acid variants with temporal trend were found to covary with other time-trending variants. The individual time trends were generally compatible, i.e. both time trends were concordant if the coupling terms were positive and discordant if the coupling terms were negative (Table 4).

**Table 4:** Direct coupling terms for specific amino acid variants at covarying positions within the HIV-1 integrase

Position *i*	Amino acid α	Position *j*	Amino acid β	e*ij* (α, β)	Time trend	Minor INSTI resistance mutation	INSTI-selected mutation
10	D	122	I	0.0683			*
10	E	122	I	−0.0753			*
11	D	119	S	−0.0940	*		
11	D	154	I	−0.0527	*		*
11	D	154	M	0.0335	*		
11	E	119	S	0.0909	*		
11	E	154	I	0.0577	*		*
11	E	154	M	−0.0368	*		
17	N	124	A	0.0690		*	
17	S	124	A	−0.0598		*	
23	A	201	I	−0.0490			*
23	A	201	V	0.0525			*
23	V	201	I	0.0432			*
23	V	201	V	−0.0443			*
31	I	154	I	−0.0438			*
31	V	154	I	0.0352			*
39	C	154	I	−0.0329			*
39	C	201	I	0.0776			*
39	C	201	V	−0.0725			*
39	S	154	I	0.0384			*
39	S	201	I	−0.0859			*
39	S	201	V	0.0828			*
72	I	265	A	0.0587	*		
72	I	265	V	−0.0612	*		
72	V	265	A	−0.0622	*		
72	V	265	V	0.0676	*		
101	I	111	K	−0.0743			*
101	I	111	T	0.0334			*
101	I	154	I	−0.0462	*		*
101	I	154	M	0.0645	*		*
101	L	154	I	0.0606	*		*
101	L	154	M	−0.0695	*		
112	A	124	A	0.0205		*	
112	I	124	A	0.0205		*	
112	I	201	I	0.0482			*
112	I	201	V	−0.0538			*
112	T	124	A	−0.0447		*	
112	T	201	I	−0.0483			*
112	T	201	V	0.0490			*
119	G	122	I	0.0576			*
119	P	122	I	0.1303			*
119	S	122	I	−0.1695	*		*
119	T	122	I	−0.0194			*
124	A	125	T	0.0266		*	
124	A	125	V	−0.0288		*	
124	A	211	K	−0.0568		*	
124	A	211	R	0.0263		*	
124	A	211	T	0.0294		*	
124	S	125	A	0.0244			*
124	T	125	A	−0.0205			*
154	I	165	I	0.0893	*		*
154	I	165	V	−0.0976	*		*
154	I	256	D	0.0333			*
154	I	256	E	−0.0369	*		*
154	I	265	A	0.0520	*		*
154	I	265	V	−0.0484	*		*
154	M	165	I	−0.1011	*		
154	M	165	V	0.1118	*		
154	M	256	E	0.0525	*		
154	M	265	A	−0.0496	*		
154	M	265	V	0.0482	*		
157	Q	160	K	−0.0632		*	
157	Q	160	Q	0.0649		*	
201	I	256	D	0.0623			*
201	I	256	E	−0.0611	*		*
201	I	265	A	0.0484	*		*
201	I	265	V	−0.0466	*		*
201	V	256	E	0.0604	*		
201	V	265	A	−0.0535	*		
201	V	265	V	0.0547	*		

Couplings with time-trending amino acid variants, minor INSTI resistance mutations, and INSTI-selected mutations are indicated by a star

Two minor INSTI resistance mutations (T124A, E157Q) and five INSTI-selected mutations (L101I, T122I, T125A, M154I, V201I) covaried with non-resistance-associated variants (Table 4).

## Discussion

Our analyses could confirm and considerably extend previously published results based on INSTI-naïve [26, 31–35] or ART-naïve [35–40] study populations that either focused on HIV-1 subtype B [31, 36, 38] or included various HIV-1 group M subtypes [26, 32–35, 37, 39, 40]. We restricted our analyses to HIV-1 subtype B strains because these are predominant in Germany [43–45] and because different HIV-1 subtypes have different consensus amino acids at some sites, which can bias the degree of variability when compared to consensus B [33, 35].

We found the highest amino acid variability determined by entropy, time trends, and direct coupling analysis within the CCD, the NTD, and between CCD and NTD. Sites important for enzymatic activity were in general conserved, however, some positions involved in binding the cellular cofactor LEDGF/p75 (sites 125, 165) and within the minimal nonspecific DNA binding region (sites 220, 230, 232, 234, 256, 265) were polymorphic with an overall variability ≥5%. Covariation between positions 125, 165, 256, or 265 and other sites was observed, and the DNA-binding site 234 covaried with the DNA-binding site 253. The most frequent substitutions were T125A, V165I, I220L, S230 N, D232E, L234I, D256E, and A265V. All of them occurred within the same biochemical class of amino acids, with the exception of T125A that represents a switch from a hydrophilic to a hydrophobic amino acid. The effect of this switch is not known and should be investigated experimentally. A time trend in frequency was observed for variants T125A, D256E, and A265V. The knowledge about the variability of the integrase should be taken into account for the design of genotypic resistance assays.

Most time trends were based on an exchange of two amino acids, however, a general diversification was observed at sites 119 and 124. 17 out of 22 amino acid variants with increasing or decreasing frequency covaried among each other. In general, we observed a concordant time trend for pairs with a positive direct coupling term and a discordant time trend for pairs with a negative direct coupling term. Exceptions to this rule were couplings between positions 154–265 and 201–256 (Table 4). The time trends for the individual variants 154I–A265, M154-265 V, and V201-256E were discordant despite positive direct coupling terms. The reason for this may be unidirectional coupling, i.e. 154I requires the presence of A265, but not vice versa. Likewise, the time trends of M154-A265, 154I-265 V, and 201I-256E were concordant despite negative direct coupling terms.

The prevailing concordance of significant time trends and significant couplings in our study suggests the selection of coevolving epistatic clusters. However, due to the transmission bottlenecks [18], genetic drift may be another viable explanation for the observed time trends in the frequency of certain amino acid variants. The role of genetic drift in HIV evolution is debated and has been quantified to some extent at the level of intra-patient evolution and for known transmission pairs rather than on inter-patient level [60, 61]. Genetic drift on inter-patient level requires inheritance of selectively neutral substitutions. Large parts of the integrase may be under negative selection to maintain enzymatic functionality; nevertheless, particular positions and certain substitutions of the integrase may be selectively neutral. Therefore, we considered the possibility of genetic drift for all time-trending substitutions by assessing whether there was evidence for (i) inheritance of the time-trending substitutions and (ii) whether the time-trending substitutions were selectively neutral. Ad (i): We statistically compared the mean patristic distance of random sequences versus the mean patristic distance of sequences carrying a specific time-trending substitution to investigate if time-trending substitutions appeared more frequently in phylogenetically closely related sequences (Additional file 1: Table S1). Sequences carrying the time-trending substitutions 72 V, 154I, 165I, and 265 V had significantly smaller mean patristic distances than random, by this indicating inheritance. Interestingly, all of these substitutions decreased in frequency over time. Ad (ii): First, we performed Tajima’s D test [60, 61] with a result of D = −1.44, by this indicating negative selection. Next, we calculated the ratio of nonsynonymous over synonymous mutations (dN/dS ratio) [60, 61], finding that most regions of the integrase were under strong negative selection, including sites 72, 154, 165, and 265 (Additional file 2: Figure S1). In summary, we could not observe a clear contribution of genetic drift to the time trends of the examined substitutions.

By using Sanger sequencing of bulk RT-PCR products with a sensitivity of approximately 30% [62, 63] and excluding ambiguous amino acids in our analyses we could only investigate the major virus variant from each patient sample. Minor variants and linkage between minor variants can only be investigated by using more sensitive techniques like single genome sequencing (SGS) or next generation sequencing (NGS) [63–65]. To minimize the probability that technical errors during RT-PCR and Sanger sequencing lead to false positive predictions with regard to coupling terms, we combined our direct coupling analysis with a power analysis, which essentially requires that an amino acid pair has to be present in multiple sequences to be repeatedly identified by direct coupling analysis. Recently, the use of covariation methods as a measure of coevolution has been questioned by Talavera et al. [66]. Based on a computational study, the authors point out that a strong covariation signal is caused by a low evolutionary rate. We therefore assessed our results accordingly but could not find a relation between the rarity of pairwise substitutions and high coupling terms or the occurrence of single substitutions in couplings (Additional file 3: Figure S2).

16 minor INSTI resistance mutations and 11 INSTI-selected mutations were observed as naturally occurring in our ART-naïve study population, which originated from the time prior to INSTI approval. Among these resistance-associated variants, three increased in frequency over time and seven covaried with non-resistance-associated variants. The complex interdependent evolution of these mutations might control enzymatic activity and replication capacity independent of selective pressure through INSTIs at the inter-patient level. Indeed, accessory drug resistance mutations that compensate viral fitness are often already polymorphic in drug-sensitive HIV-1, suggesting that these mutations may naturally enhance viral fitness and virulence with progression of the HIV-1 epidemic [21, 22]. INSTI-independent linkage between non-resistance-associated sites and resistance-associated sites or sites targeted by INSTIs can affect the selection of resistance mutations in the presence of INSTIs. This knowledge should be taken into account for the improvement of resistance prediction algorithms as well as for the development and preclinical evaluation of new INSTIs and ALLINIs. Deeper analyses of the observed resistance-associated variants are needed to evaluate their clinical relevance. In particular, those with naturally increasing frequencies that were linked to covariation should be investigated, i.e. L101I, T122I, and V201I. The absence of major INSTI resistance mutations in our ART-naïve study population underscores the suitability of INSTIs for first-line antiretroviral regimens.

Because the analysed dataset was rather small (*n* = 337), our results may require further validation from the analysis of larger, independent datasets. Due to the relatively small number of samples, some of our results might not have reached statistical significance, e.g. the temporal trend of T122 and D256. Generally, given the small sample size, overrepresentation of almost identical sequences (i.e. from transmission chains) may profoundly bias any downstream analysis of time trends and covariation patterns. To assess whether our analyses were affected by such sampling bias, we additionally performed them using a reduced dataset in which clusters of closely related sequences were replaced by one representative only. The results obtained from the reduced dataset confirmed all results obtained from the full dataset.

## Conclusions

Our aim was to analyse the molecular evolution of the HIV-1 integrase prior to the approval of INSTIs and, thus, INSTI selective pressure at the inter-patient level. We found significant time trends in the frequency of certain amino acid variants, suggesting ongoing adaptation of the enzyme. Upon closer inspection, we found that amino acid variants with significant time trends covaried with other time-trending variants. Such a linkage may impose constraints that determine the evolutionary trajectory of the integrase and that influence the selection of INSTI resistance mutations. Our results can help in evaluating the resistance potential of naturally occurring polymorphisms and in understanding the development of resistance in the presence of INSTIs.

## Additional files

Additional file 1: Table S1. Statistics assessing whether time-trending substitution could have resulted from inheritance. (DOCX 11 kb)

Additional file 2: Figure S1. dN/dS ratio for different regions of the HIV-1 integrase. The red line depicts the median and the grey shaded area the 25 to 75 percentile range. Black dots mark time-trending positions, i.e. codons 6, 11, 72, 101, 119, 122, 124, 135, 154, 165, 201, 256, 265. A sliding window with window size 5 was applied. (EPS 542 kb)

Additional file 3: Figure S2. Relation between significant coupling and frequency of single/pairwise substitutions. a) Relation between the relative frequencies of substitution pairs within the MSA and the strength of their coupling values (only significant couplings are considered). b) Relation between the relative frequencies of single substitutions within the MSA and their absolute frequency in significant couplings. (ZIP 133 kb)
