# Supplementary material for: Molecular evolution of HIV-1 integrase during the 20 years prior to the first approval of integrase inhibitors
Source: Virol J. 2017 Nov 14;14:223. doi: 10.1186/s12985-017-0887-1 (PMC5686839; doi:10.1186/s12985-017-0887-1)
Supplement: Supplementary file 1 — Statistics assessing whether time-trending substitution could have resulted from inheritance. (DOCX 11 kb) [file 12985_2017_887_MOESM1_ESM.docx]

**Table S1: Statistics assessing whether time-trending substitution could have resulted from inheritance.**

| **Substitution** | ***P* value** |
| --- | --- |
| 6E | 0.9984 |
| 11D | 1 |
| 72V | 0.0274 |
| 101I | 1 |
| 122I | 1 |
| 135V | 1 |
| 154I | 0 |
| 165I | 0 |
| 201I | 1 |
| 256E | 1 |
| 265V | 0 |

Small *P* values indicate that substitutions are significantly more closely related than random, i.e. they are clustered in the phylogenetic tree and there is evidence for inheritance. *P* values were calculated against a null model. For a particular substitution, we drew N random sequences, matching the number of sequences bearing the particular time-trending substitution. For these N random sequences, we computed the mean patristic distance μ_rand_. The procedure was repeated M=10000 times to obtain a statistic for the mean patristic distance of N random sequences. We then computed the patristic distance of sequences harbouring the time-trending substitution μ_subst_. To compute a *P* value assessing whether the mean patristic distance of sequences with the time-trending substitution was smaller than the mean patristic distance of the random sequences (H0: μ_subst_ ≥ μ_rand_; H1: μ_subst_ < μ_rand_), we counted *P* = (#μ_subst_ ≥ μ_rand_)/M, where ‘#μ_subst_ ≥ μ_rand_’ denotes the number of times where the null hypothesis was true.
